# Comparative Risk of Recurrent Esophageal Variceal Hemorrhage and Other Decompensation Events with Carvedilol Versus Propranolol in Patients with Cirrhosis: A Retrospective Study

**DOI:** 10.1007/s10620-025-09453-6

**Published:** 2025-10-18

**Authors:** Abdellatif Ismail, Mohammed Abusuliman, Mohammad Kloub, Shahem Abbarh, Khalid Aloum, Mostafa Suhail Najim, Mohammed Al-Aquily, Mahmoud Y. Madi, Kamran Qureshi, Wing-Kin Syn

**Affiliations:** 1 Division of Gastroenterology and Hepatology, Digestive Disease and Surgery Institute, Cleveland Clinic-Florida, 2950 Cleveland Clinic Blvd., Weston, FL 33311 USA; 2 Department of Internal Medicine, Henry Ford Hospital, Detroit, MI USA; 3 Department of Internal Medicine, New York Medical College - Saint Michael’s Medical Center, Newark, NJ USA; 4 Department of Internal Medicine, MedStar Health, Baltimore, USA; 5 Division of Gastroenterology and Hepatology, Colorado University, Denver, CO USA; 6 Department of Internal Medicine, Rochester Regional Health - Unity Hospital, Rochester, NY USA; 7 Department of Internal Medicine, Norwalk Hospital/Yale University Program, Norwalk, CT USA; 8 Division of Gastroenterology and Hepatology, University of Texas at Austin Dell Medical School, Austin, USA; 9 Division of Gastroenterology and Hepatology, Department of Internal Medicine, Saint Louis University School of Medicine, St. Louis, MO USA; 10 Department of Physiology, Faculty of Medicine and Nursing, University of Basque Country UPV/EHU, Viscaya, Spain

**Keywords:** Decompensated cirrhosis, Gastrointestinal bleeding, Hepatic encephalopathy, Ascites, Portal hypertension, Beta-blockers

## Abstract

**Background & Aims:**

Non-selective beta-blockers (NSBBs) have become a cornerstone treatment to prevent complications of cirrhosis and portal hypertension. Data supporting the use of a specific beta-blocker are scarce. In this retrospective study, we aimed to compare the effectiveness of carvedilol versus propranolol in: 1) preventing recurrent esophageal hemorrhage (EVH) in patients with prior history of EVH 2) reducing the occurrence of further decompensation episodes in these patients including hepatic encephalopathy (HE), ascites, spontaneous bacterial peritonitis (SBP), hepatorenal syndrome (HRS), and hepatocellular carcinoma (HCC), and 3) reducing all-cause mortality.

**Approach & Results:**

This was a retrospective propensity-matched study using the multi-institutional database TriNetX. We included patients with cirrhosis who had an episode of EVH and were prescribed carvedilol or propranolol between December 2004 and December 2024. The primary outcome was the rate of hospitalization with recurrent EVH within the first 5 years of starting the index NSBB. The secondary outcomes were hospitalization with the principal diagnoses of ascites, SBP, HE, HRS, new diagnosis of HCC, undergoing liver transplant (LT), and all-cause mortality within the observation period of 5 years of NSBB prescription. Kaplan–Meier survival analysis was also performed. Compared to propranolol use, carvedilol use was associated with lower risk of EVH (RR, 0.898, *P* < 0.001), ascites (RR, 0.757, *P* < 0.001), SBP (RR, 0.680, *P* < 0.001), HRS (RR, 0.734, *P* < 0.001), HCC (RR, 0.701, *P* < 0.001), undergoing LT (RR, 0.825, *P* = 0.028) and mortality (RR, 0.640, *P* < 0.001). No difference in HE rates (RR, 0.899, *P* = 0.071) was found between the two groups.

**Conclusions:**

Compared to propranolol, the use of carvedilol in patients with history of cirrhosis and EVH was associated with lower risk of recurrent EVH, further decompensation episodes, undergoing LT, and mortality.

**Supplementary Information:**

The online version contains supplementary material available at 10.1007/s10620-025-09453-6.

## Introduction

Liver cirrhosis is a non-reversible condition characterized by extensive scarring of the liver that replaces normal tissue, reducing the amount of functional hepatocytes and distorting its architecture [1]. The leading causes of liver cirrhosis are alcohol-related liver disease (ALD), hepatitis C and B virus infections, and Metabolic dysfunction-associated Steatotic Liver Disease (MASLD) [1]. Patients with liver cirrhosis are broadly categorized as being in one of two different stages: compensated—an early stage in which the liver still functions despite the parenchymal damage [2]; and decompensated—an advanced stage in which the liver cannot perform the crucial hepatic functions of detoxification, protein production, lipid and cholesterol metabolism [3]. Decompensated liver cirrhosis is defined by one or more of the following manifestations; ascites, hepatic encephalopathy (HE), esophageal variceal hemorrhage (EVH), and hepatocellular carcinoma (HCC) [4].

A major pathophysiological process seen in liver cirrhosis is portal hypertension, which results in blood shunting from the portal to the systemic circulation through portosystemic collateral pathways including esophageal, gastric, and rectal venous beds [5]. This eventually results in excessive distension in the systemic collateral venous beds and renders them prone to rupture and bleeding, creating what is known as varices. The most important location of varices formation is esophageal varices which have a high potential for massive gastrointestinal (GI) bleeding upon rupture. Portal hypertension also plays a major role in the development of non-bleeding cirrhosis complications namely; ascites, spontaneous bacterial peritonitis (SBP), hepatorenal syndrome (HRS), and HE [6]. A clinical trial done in 1981 that evaluated the non-selective beta-blocker (NSBB) propranolol for patients with cirrhosis and portal hypertension became a landmark study after showing that this treatment was associated with decreased risk of EVH [7]. Since that study, NSBBs have become cornerstone treatments to prevent complications of cirrhosis, such as upper GI bleeding, HE, ascites, and mortality [8]. Robust evidence suggests NSBBs over no therapy or esophageal variceal ligation (EVL) alone to prevent variceal bleeding and improve survival in compensated liver cirrhosis patients [9–12].

NSBBs work by non-selectively blocking beta-1 and beta-2 adrenergic receptors, resulting in reduced cardiac output and splanchnic vasoconstriction, respectively, and a reduction in portal venous pressure and hepatic venous pressure gradient (HVPG) [13–15]. Carvedilol has the additional effects of blockage of alpha-1 adrenergic receptors, release of nitric oxide, and intrahepatic vasodilation, resulting in a more robust reduction in HVPG compared to classic NSBBs (cNSBBs) such as propranolol and nadolol [14, 16, 17]. Evidence supporting the use of carvedilol versus other cNSBBs for secondary prophylaxis for EVH has been rapidly growing. [18–22]. Meanwhile, some studies [23, 24] suggested deleterious effects on renal function and survival with carvedilol use in patients with cirrhosis and attributed that to stronger effects on cardiac output (CO) and mean arterial pressure (MAP).

Therefore, we performed a retrospective study to compare the rates of recurrent EVH and other decompensation episodes in patients with cirrhosis and a history of EVH within 5 years of being prescribed carvedilol or propranolol. Our primary aim was to compare the effectiveness of these two NSBBs in preventing recurrent EVH (secondary prophylaxis). We additionally assessed the comparative effectiveness of these two NSBBs in reducing other non-bleeding decompensation events, including ascites, SBP, HE, HRS, HCC, and reducing the rates of mortality.

## Materials and Methods

### Design and Data Source

This was a retrospective propensity-matched comparative effectiveness study of patients with cirrhosis and a history of EVH who were prescribed the cNSBB propranolol or carvedilol. Data were obtained from the multi-institutional database TriNetX (Cambridge, MA, US) United States (US) Collaborative Network, which is a global federated research network that provides real-time access to electronic health records of about 213 million patients within 92 healthcare organizations in the US. All data were derived from the electronic health records and through a built-in natural language processing system that extracts variables from clinical documents. The TriNetX interface provides only aggregate counts and statistical summaries to protect patient health information, ensuring the data remains de-identified at all levels.

### Study Population

A real-time search of the US Collaborative Network in the TriNetX platform was conducted to extract clinical data for adult patients (≥ 18 years old) with cirrhosis who had an episode of EVH and were prescribed carvedilol or propranolol between December 2004 and December 2024. The start time was chosen as TrinetX limits its search to data within 20 years. TrinetX allows establishing temporality between different groups within a cohort, and we limited our search to patients who had the NSBB started after EVH diagnosis. We used the following International Classification of Disease, Tenth Revision, and Clinical Modification (ICD-10-CM) codes to identify patients with cirrhosis: K74.6, K70.3, and the ICD-10-CM codes I85.01 and I85.11 to identify a history of EVH. The cohort was then stratified into 2 groups: patients who were prescribed carvedilol and those who were prescribed propranolol after EVH diagnosis. We used the RxNorm code 20352 to identify carvedilol and 8787 to identify propranolol. TrinetX allows medication strength identification, and we limited our search to patients started on the recommended daily dose of carvedilol for portal hypertension of 6.25 mg or 12.5 mg [14].

Additionally, we created a third group consisting of patients who did not receive any beta-blockers (no beta-blocker group) and compared their outcomes to those in the carvedilol group in a separate (second) analysis.

### Outcomes

The primary outcome was the rate of hospitalization with recurrent EVH within the first 5 years of starting the index NSBB. The secondary outcomes were hospitalization with the principal diagnoses of ascites, SBP, HE, HRS, & new diagnosis of HCC, receiving LT, and mortality within the observation period. Episodes that occurred before the index medications were excluded from the analysis. Outcomes were identified per the following ICD-10-CM codes: ICD-10 codes I85.01 & I85.11 for EVH; ICD-10 code K76.82 for HE; ICD-10 code R18 for ascites; ICD-10 code K65.2 for SBP; ICD-10 code K76.7 for HRS; ICD-10 code C22.0 for HCC; & ICD-10 code Z94.4 for LT status, Supplemental Table 1.

For Kaplan–Meier survival analysis, the timeline was defined starting from the index event date (starting NSBB), with patients being followed longitudinally until the occurrence of the outcome or until they were censored at their last recorded follow-up. This approach allowed the analysis of time-to-event data, estimating the cumulative incidence of outcomes over time.

### Statistical Analysis

The primary analysis evaluated two groups: patients who were prescribed carvedilol (6.25 mg or 12.5 mg) and patients who were prescribed propranolol. All statistical analyses were conducted in the TriNetX software with the browser-based real-time analytics feature, TriNetx Live (TriNetX LLC, Cambridge, MA). Baseline characteristics of all groups were described with means ± standard deviation for continuous data and counts and percentages for categorical data. Covariates based on demographics, comorbid diseases, prior procedures, and medications were identified. Comparison groups were matched per a one-to-one (1:1) PSM strategy to balance the following covariates between them: age, sex, race, body mass index (BMI), presence of comorbidities (acute kidney injury, chronic kidney disease, alcohol use disorder, HE, ascites, SBP, and HRS, prior esophageal variceal ligation (EVL), medications (diuretics, antibiotics, antilipemic agents, and sedatives), and baseline laboratory tests (sodium, creatinine, albumin, total bilirubin, international normalized ratio (INR) [elements of Model for End-Stage Liver Disease [MELD] score], hemoglobin, and platelet count). These covariates were chosen because they were either significantly different between the comparator groups or because they were known to affect the risk of recurrent EVH or one or more of the other outcomes. The TriNetX platform uses input matrices of the user-identified covariates to conduct logistic regression analysis to obtain propensity scores for all subjects. The propensity scores generated were used to match patients using greedy nearest-neighbor algorithms with a caliper width of 0.1 pooled standard deviations. TriNetX randomizes the order of rows to eliminate bias resulting from nearest-neighbor algorithms. The propensity score matching aimed to account for differences in baseline characteristics that could affect the outcomes of interest. After PSM, the risk of each outcome was calculated and expressed as the relative risk (RR) with 95% confidence intervals (CI). Kaplan–Meier survival curves were generated to visually represent the time-to-event for each of the outcomes between the two groups. The log-rank test was applied to assess the statistical significance of differences between the curves. A two-sided p-value < 0.05 was considered statistically significant for all analyses.

Moreover, a second analysis was performed to compare the EVH recurrence and mortality between the carvedilol group and the third group (no beta-blocker).

## Results

### Baseline Characteristics

**Fig. 1:**
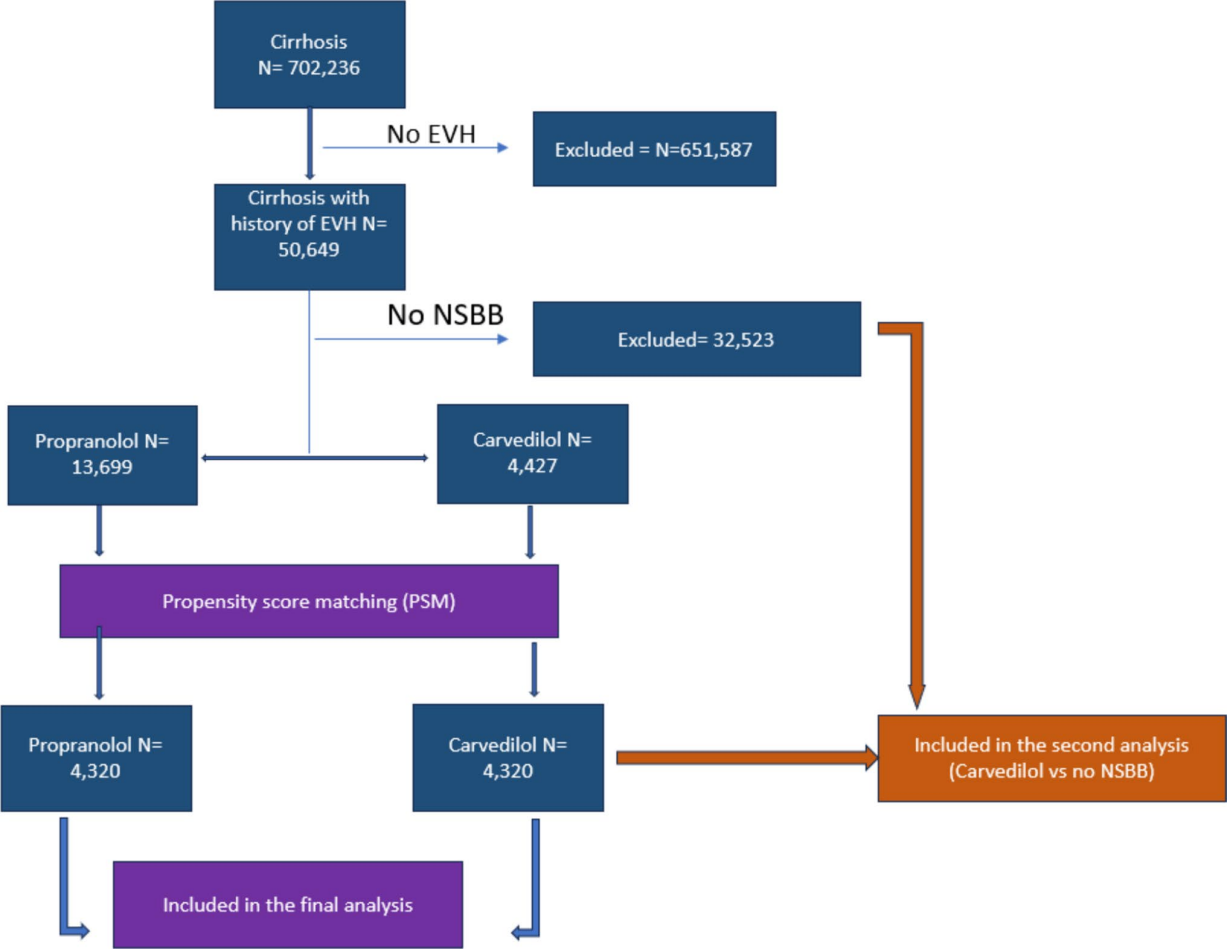
Flowchart of study cohorts. *EVH* Esophageal Variceal Hemorrhage, *NSBB* Non-selective Beta-Blockers, *N* number

We identified 702,236 patients diagnosed with cirrhosis and subsequently 50,649 patients with cirrhosis and EVH excluding patients without a history of EVH (*N* = 651,587). Among the included patients, we identified 4,427 patients who were started on carvedilol (carvedilol group) and 13,699 patients who were started on propranolol (propranolol group), excluding 32,523 patients who were not started on any NSBB. After PSM, 4,320 patients were included in each of the carvedilol and propranolol groups and were included in the final analysis. Additionally, we created a third group that consisted of patients who were not on beta-blockers (no beta-blockers group, (*n* = 32,523) and included it in a second analysis, Fig. 1.

After PSM, the mean age was similar between the two matched groups (58.8 ± 12.4 years for carvedilol versus 59.0 ± 11.8 years for propranolol; *P* = 0.972). The proportion of male patients was also comparable after matching (63.7%% for carvedilol versus 63.2% for propranolol; *P* = 0.639). Similarly, racial distributions were well-balanced between the two groups. Furthermore, we matched the two groups for the elements of MELD score to match for disease severity (Table 1). Finally, we matched for history of EVL and after PSM, 46.5% of patients in the carvedilol group vs. 47.2% in the propranolol group had history of EVL.

**Table 1:** Demographic and clinical characteristics of patients in the comparator groups before and after PSM

	Before propensity matching			After propensity matching		
	Carvedilol *N* = 4427	propranolol *N* = 13,699	*P*-value	Carvedilol *N* = 4320	propranolol *N* = 4320	*P*-value
Age at index (years, mean ± SD)	59.0 ± 12.4	55.4 ± 11.9	< 0.001	58.8 ± 12.4	58.8 ± 11.8	0.972
Male sex	63.5%	65.0%	0.069	63.7%	63.2%	0.639
Race, %						
Caucasian	74.6%	71.4%	< 0.001	74.5%	74.6%	0.902
African American	8.2%	6.0%	< 0.001	8.1%	8.1%	0. 968
Hispanic	13.0%	20.0%	< 0.001	13.2%	13.0%	0.774
Comorbidities & complications, %						
Hepatic encephalopathy	26.1%	15.6%	< 0.001	25.2%	26.0%	0.402
Ascites	57.1%	50.8%	< 0.001	56.6%	56.4%	0.828
Spontaneous bacterial peritonitis	8.7%	7.6%	0.011	8.7%	8.2%	0.439
Hepatorenal syndrome	8.1%	4.7%	< 0.001	7.6%	7.5%	0.903
Alcohol use disorder	43.9%	48.3%	< 0.001	44.3%	43.0%	0.233
AKI and CKD	48.6%	31.2%	< 0.001	47.5%	47.6%	0.966
Antibiotics, %						
Ciprofloxacin	35.3%	27.7%	< 0.001	34.6%	34.0%	0.587
Norfloxacin	0.8%	0.4%	0.009	0.7%	0.7%	0.799
Moxifloxacin	4.9%	2.8%	< 0.001	4.6%	4.5%	0.918
Ceftriaxone	59.5%	49.3%	< 0.001	58.8%	58.3%	0.585
Piperacillin/tazobactam	20.8%	14.7%	< 0.001	20.3%	19.0%	0.137
Trimethoprim/Sulfamethoxazole	24.9%	13.2%	< 0.001	23.4%	23.2%	0.799
Procedures, %						
Esophageal Band Ligation	47.0%	38.3%	< 0.001	46.5%	47.2%	0.532
TIPS	6.3%	3.0%	< 0.001	5.7%	5.3%	0.322
Injection sclerosis	0.8%	0.8%	0.967	0.8%	0.8%	0.905
Others						
BMI (kg/m^2^)	29.4 ± 6.7	28.7 ± 6.6	< 0.001	29.4 ± 6.7	29.1 ± 6.7	0.033
HR (beats/min)	78.2 ± 15.6	78.0 ± 15.9	0.502	78.3 ± 15.7	77.3 ± 15.6	0.024
Sodium (mmol/L)	137.6 ± 4.2	137.2 ± 4.5	< 0.001	137.6 ± 4.2	137.3 ± 4.7	0.010
Creatinine (mg/dl)	1.2 ± 1.1	1.0 ± 1.6	< 0.001	1.1 ± 1.1	1.1 ± 2.4	0.944
Hemoglobin (g/dl)	10.1 ± 2.4	9.9 ± 2.5	0.001	10.1 ± 2.4	9.8 ± 2.4	< 0.001
Platelets (K/mcL)	115.3 ± 76.1	103.9 ± 67.3	< 0.001	114.6 ± 75.5	105.8 ± 68.9	< 0.001
Total Bilirubin (mg/dl)	2.0 ± 3.0	2.9 ± 4.2	< 0.001	2.0 ± 3.0	2.6 ± 3.7	< 0.001
Albumin (g/dl)	3.2 ± 0.7	3.0 ± 0.7	< 0.001	3.2 ± 0.7	3.0 ± 0.7	< 0.001
INR	1.3 ± 0.3	1.4 ± 0.4	< 0.001	1.3 ± 0.3	1.4 ± 0.4	< 0.001

*AKI* acute kidney injury, *CKD* chronic kidney disease, *SD* standard deviation, *BMI* Body Mass Index, *INR* International Normalized Ratio, *PSM* propensity score matching, *TIPS* transvenous intrahepatic portosystemic shunt, *HR* Heart Rate

### Clinical Outcomes

#### Recurrent Esophageal Variceal Hemorrhage (EVH)

**Fig. 2:**
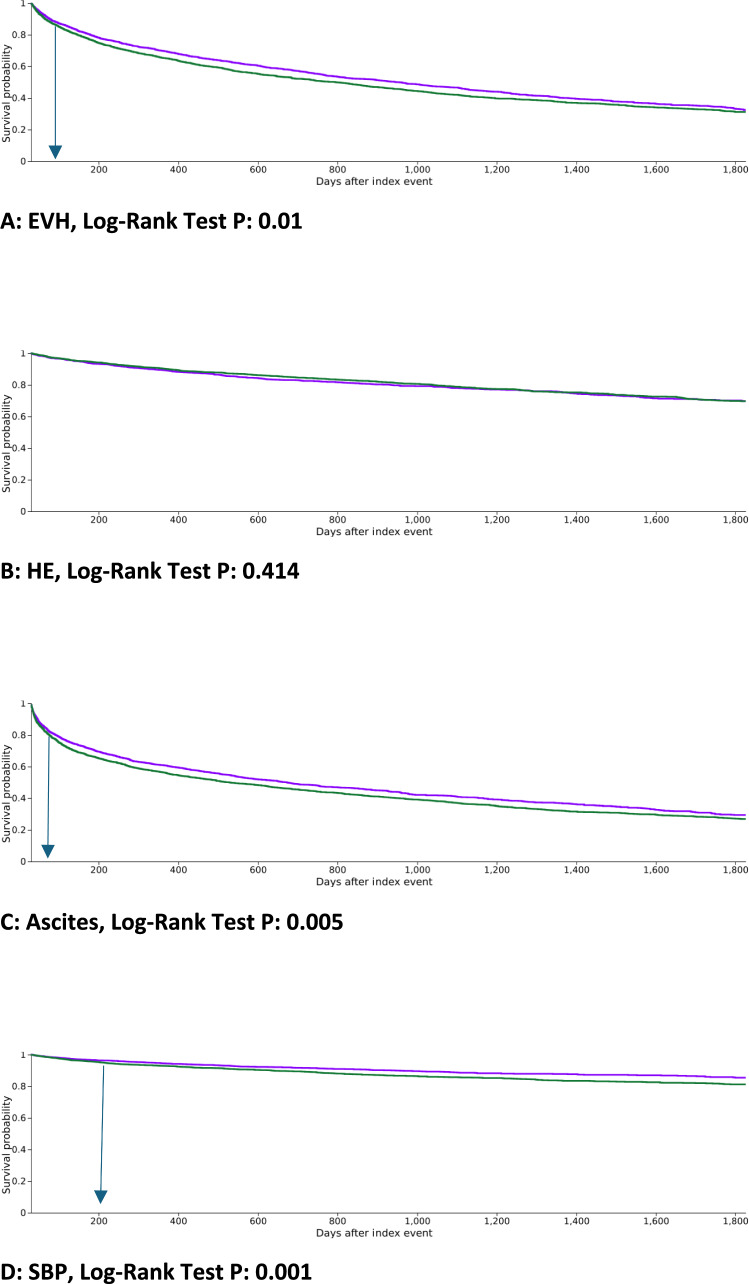

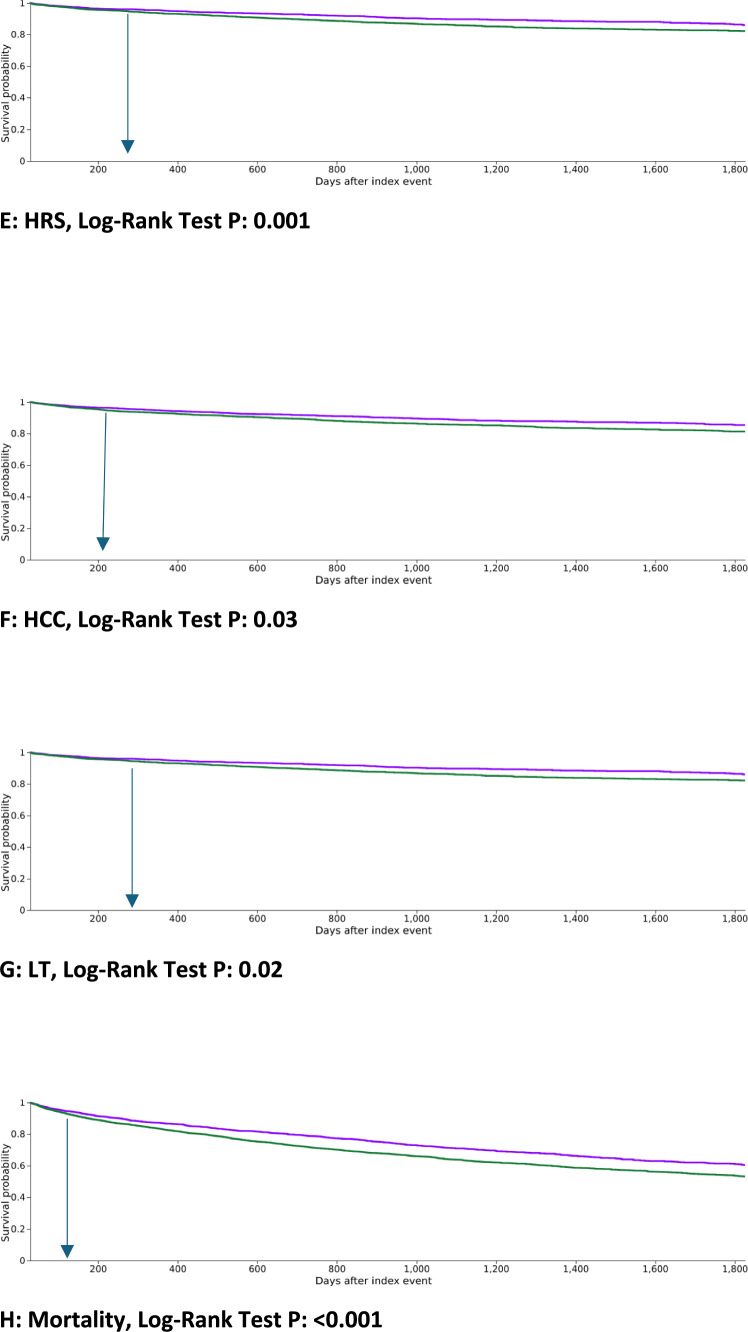
Kaplan–Meier Survival analysis. Proportion of event-free patients and time-to-event analysis (in days). Index event: starting the NSBB (carvedilol or propranolol). **A**: EVH, **B**: HE, **C**: Ascites, **D**: SBP, **E**: HRS, **F**: Mortality. *EVH* Esophageal Variceal Hemorrhage; *NSBB* Non-selective Beta-Blockers *SBP* Spontaneous Bacterial Peritonitis *HE* Hepatic Encephalopathy, *HRS* Hepatorenal Syndrome, *LT* Liver Transplant

A total of 1377 patients (31.9%) in the carvedilol group experienced an episode of recurrent EVH during the observation period compared to 1534 patients (35.5%) in the propranolol group. Patients who had the outcome prior to the time window were excluded. The risk of EVH was slightly lower in the carvedilol group (RR, 0.898; 95% CI, 0.846–0.952; *P* < 0.001) (Table 2), reflecting a superior, albeit small, benefit of carvedilol for secondary prophylaxis of EVH. Kaplan–Meier analysis showed a significantly lower cumulative incidence of EVH in the carvedilol group than in the propranolol group (Log-Rank Test P: 0.01). It demonstrated that the risk of EVH increased more rapidly in the propranolol group, compared to the carvedilol group, after about 60 days of starting the index NSBB, with a divergence in curves over time. The median time to EVH was 1000 days in the carvedilol group and 850 days in the propranolol group (Fig. 2A).

**Table 2:** Risk of recurrent esophageal variceal hemorrhage and other decompensation events in patients with liver cirrhosis taking carvedilol versus propranolol

Outcome	*N* (%) with outcome after PSM	RR (95% CI)	*P*-value
Esophageal variceal hemorrhage			
Carvedilol	1377 (31.9)	0.898 (0.846, 0.952)	< 0.001
Propranolol	1534 (35.5)	Ref	
Hepatic encephalopathy			
Carvedilol	463 (14.9)	0.899 (0.801, 1.009)	0.071
Propranolol	509 (16.6)	Ref	
Ascite			
Carvedilol	419 (25.3)	0.757 (0.680, 0.843)	< 0.001
Propranolol	554 (33.4)	Ref	
Spontaneous bacterial peritonitis			
Carvedilol	209 (5.4)	0.680 (0.574, 0.807)	< 0.001
Propranolol	308 (7.9)	Ref	
Hepatorenal syndrome			
Carvedilol	232 (5.9)	0.734 (0.623, 0.865)	< 0.001
Propranolol	314 (8.0)	Ref	
Mortality			
Carvedilol	839 (20.2)	0.640 (0.593, 0.690)	< 0.001
Propranolol	1282 (31.6)	Ref	
HCC			
Carvedilol	165 (4.3)	0.701 (0.578, 0.852)	< 0.001
Propranolol	234 (6.2)	Ref	
LT			
Carvedilol	218 (5.7)	0.825 (0.695, 0.980)	0.028
Propranolol	280 (6.9)	Ref	

*CI* confidence interval, *PSM* propensity score matching, *RR* risk ratio, *UGIB* Upper Gastrointestinal Bleeding, *HCC* Hepatocellular carcinoma, *LT* Liver Transplant

#### Hepatic Encephalopathy (HE)

New or worsening HE occurred in 463 patients (14.9%) in the carvedilol group and 509 patients (16.6%) in the propranolol group during the observation period. Patients who had the outcome prior to the time window were excluded. No significant difference in the risk of HE between the two groups was seen (RR, 0.899; 95% CI, 0.801–1.009; *P* = 0.0.71) and no significant difference on the Kaplan–Meier analysis was observed either, Log-Rank Test *P*: 0.414 (Table 2), (Fig. 2B).

#### Ascites

New or worsening ascites occurred in 419 patients (25.3%) in the carvedilol group and 554 patients (33.4%) in the propranolol group, indicating a lower risk of ascites in the carvedilol group (RR, 0.757; 95% CI, 0.680–0.843; *P* < 0.001). Patients who had the outcome prior to the time window were excluded. Kaplan–Meier analysis showed a significantly lower cumulative incidence of ascites in the carvedilol group than in the propranolol group (Log-Rank Test P: 0.005). It demonstrated that the risk of ascites increased more rapidly in the propranolol group, compared to the carvedilol group, after about 50 days of starting the index NSBB, with a divergence in curves over time. The median time to ascites outcome was 673 days in the carvedilol group and 530 days in the propranolol group (Table 2), (Fig. 2C).

#### Spontaneous Bacterial Peritonitis (SBP)

A total of 209 patients (5.4%) in the carvedilol group experienced an episode of SBP compared to 308 patients (7.9%) in the propranolol group, revealing a significantly lower risk of SBP in patients on carvedilol (RR, 0.680; 95% CI, 0.574–0.807; *P* < 0.001). Patients who had the outcome prior to the time window were excluded. Kaplan–Meier analysis showed a significantly lower cumulative incidence of SBP in the carvedilol group than in the propranolol group (Log-Rank Test P: 0.001). It demonstrated that the risk of SBP increased more rapidly in the propranolol group, compared to the carvedilol group, after around 200 days of starting the index NSBB, with a divergence in curves over time (Table 2), (Fig. 2D).

#### Hepatorenal Syndrome (HRS)

A total of 232 patients (5.9%) in the carvedilol cohort experienced an episode of HRS compared to 314 patients (8%) in the propranolol cohort, revealing a significantly lower risk of HRS in patients on carvedilol (RR, 0.734; 95% CI, 0.623–0.865; *P* < 0.001). Patients who had the outcome prior to the time window were excluded. Kaplan–Meier analysis showed a significantly lower cumulative incidence of HRS in the carvedilol group than in the propranolol group (Log-Rank Test P: 0.001). It demonstrated that the risk of HRS increased more rapidly in the propranolol group, compared to the carvedilol group, after around 300 days of starting the index NSBB, with a divergence in curves over time (Table 2), (Fig. 2E).

#### Hepatocellular Carcinoma (HCC)

A total of 165 patients (4.3%) in the carvedilol cohort had a new diagnosis of HCC compared to 234 patients (6.2%) in the propranolol cohort, revealing a significantly lower risk of HCC in patients on carvedilol (RR, 0.701; 95% CI, 0.578–0.852; *P* < 0.001). Patients who had the outcome prior to the time window were excluded. Kaplan–Meier analysis showed a significantly lower cumulative incidence of HCC in the carvedilol group than in the propranolol group (Log-Rank Test P: 0.03). It demonstrated that the risk of HCC increased more rapidly in the propranolol group, compared to the carvedilol group, after around 200 days of starting the index NSBB, with a divergence in curves over time (Table 2), (Fig. 2F).

#### Liver Transplant (LT)

A total of 218 patients (5.7%) in the carvedilol cohort received LT compared to 280 patients (6.9%) in the propranolol cohort (RR, 0.825; 95% CI, 0.695–0.980; *P* = 0.028). Patients who had the outcome prior to the time window were excluded. Kaplan–Meier analysis showed a significantly lower cumulative rates of LT in the carvedilol group than in the propranolol group (Log-Rank Test *P*: 0.02). It demonstrated that the outcome of LT increased more rapidly in the propranolol group, compared to the carvedilol group, after around 300 days of starting the index NSBB, with a divergence in curves over time (Table 2), (Fig. 2G).

#### Mortality

Mortality rates were significantly lower in the carvedilol group, with 839 deaths (20.2%) compared to 1282 deaths (31.6%) in the propranolol group during the observation period (RR, 0.640; 95% CI, 0.593–0.690; *P* < 0.001). Kaplan–Meier analysis showed a significantly lower cumulative incidence of mortality in the carvedilol group than in the propranolol group (Log-Rank Test *P* < 0.001). It demonstrated that the risk of mortality increased more rapidly in the propranolol group, compared to the carvedilol group, after 100 days of starting the index NSBB, with a divergence in curves over time (Table 2), (Fig. 2H).

#### Second Analysis

As mentioned above, we identified 4,427 patients who were started on carvedilol (carvedilol group) and 32,523 patients who were not started on NSBB (no beta-blocker group). The two groups were well matched after PSM, and the risk of each outcome was calculated and expressed as the relative risk (RR) with 95% confidence intervals (CI). A total of 4,259 patients were included in each of these groups and were included in the second analysis. A total of 1,365 patients (32%) in the carvedilol group experienced an episode of recurrent EVH during the observation period compared to 1,856 patients (43.5%) in the no beta-blocker group. The risk of EVH was significantly lower in the carvedilol group (RR, 0.735; 95% CI, 0.680–0.854; *P* = 0.007) (Table 3), reflecting a significant benefit of carvedilol for secondary prophylaxis of EVH. Moreover, mortality rates were significantly lower in the carvedilol group, with 820 deaths (20%) compared to 1331 deaths (36.2%) in the no beta-blocker group during the observation period (RR, 0.552; 95% CI, 0.512–0.595; *P* < 0.001), reflecting a significant mortality benefit of carvedilol in patients with cirrhosis and history of EVH, (Table 3).

**Table 3:** Risk of recurrent esophageal variceal hemorrhage and other decompensation events in patients with liver cirrhosis taking carvedilol versus not using beta-blockers

Outcome	*N* (%) with Outcome after PSM	RR (95% CI)	*P*-value
Esophageal variceal hemorrhage			
Carvedilol	1365 (32.0)	0.735 (0.680, 0.854)	0.007
No BB	1856 (43.5)	Ref	
Mortality			
Carvedilol	820 (20.0)	0.552 (0.512, 0.595)	< 0.001
No BB	1331 (36.2)	Ref	

*CI* confidence interval, *PSM* propensity score matching, *RR* risk ratio, *UGIB* Upper Gastrointestinal Bleeding, *BB* Beta-blockers, *HCC* Hepatocellular carcinoma, *LT* Liver Transplant

## Discussion

In this retrospective comparative effectiveness study, we observed that patients with cirrhosis and a history of EVH who were on the NSBB carvedilol had a lower rate of recurrent EVH compared to patients prescribed propranolol. Notably, patients on carvedilol also demonstrated significantly lower rates of further decompensation episodes of ascites, SBP, HRS, HCC, and lower all-cause mortality. Patients in the carvedilol group also had lower rates of LT during the observation period. Furthermore, compared to patients not on beta-blockers, patients on carvedilol had significantly lower rates of recurrent EVH and mortality. Of note, the lower risk of recurrent EVH and ascites in the carvedilol group, compared to the propranolol group, was noted to start around 50–60 days from starting the index NSBB; while the lower risk of SBP, HCC and mortality started around 200 days, and HRS and LT around 300 days of initiating the index NSBB.

The term “further decompensation” refers to the phenomenon of developing successive complications of cirrhosis and has been associated with significantly increased mortality rates [25]. While NSBBs are currently a key therapy for patients with cirrhosis, initial studies have raised questions about their potential to exacerbate systemic hypotension and renal dysfunction, particularly in patients who have decompensated disease [23, 26]. For example, NSBBs were thought to impair cardiac output and blunt compensatory responses to portal hypertension, potentially increasing the risk of adverse outcomes in critically ill patients with cirrhosis [27]. Yet despite these initial concerns, evolving evidence has clarified the role of NSBB within the context of liver disease, including their safety and efficacy in managing portal hypertension and its complications [9].

Currently, NSBBs are regarded as an essential therapy for managing cirrhosis, and their use is supported by guidelines from leading organizations such as the American Association for the Study of Liver Diseases (AASLD) [14], the European Association for the Study of the Liver (EASL) [28], and the Baveno VII consensus [15]. However, the various guidelines contain some inconsistencies regarding the choice of specific NSBB for various patients. Notably, carvedilol is recognized for its added alpha-1 adrenergic blocking activity, which provides an additional reduction in portal pressure and improves vascular dysfunction compared to cNSBBs such as propranolol or nadolol [17, 29, 30].

The 2018 EASL practice guidelines [28] generally recommend NSBBs—but without specifying any particular agent—for primary or secondary prophylaxis of EVH in cases of medium-large varices, small varices with red wale marks, or small varices in patients with Child–Pugh C scores. These guidelines also advise caution when prescribing NSBBs in cases of severe or refractory ascites, and in particular do not recommend carvedilol for this group of patients due to the potential deleterious effect on hemodynamics. On the other hand, according to the 2022 Baveno VII consensus [15], carvedilol is the preferred NSBB to prevent decompensation in patients with compensated cirrhosis. However, inadequate evidence was available at the time of the consensus to support either carvedilol or other cNSBBs for preventing further decompensation in patients who already have a history of EVH. More recently, the 2024 AASLD Practice Guidance on risk stratification and management of portal hypertension and varices in cirrhosis [14] recommended carvedilol as the preferred NSBB for treating portal hypertension in patients with cirrhosis. This recommendation was based on its greater reduction of portal pressure in head-to-head comparisons with cNSBBs [19, 31], better tolerance, the possibility of preventing ascites, and potential mortality benefits. Our study adds to the mounting evidence supporting the beneficial effect of carvedilol in patients with cirrhosis and EVH.

Of note, a 2018 Cochrane systematic review and meta-analysis of 10 randomized clinical trials that compared carvedilol versus cNSBBs in adults with cirrhosis and esophageal varices [19] showed no clear beneficial or harmful effects of carvedilol versus cNSBBs on mortality, upper GI bleeding, or serious or non‐serious adverse events, even though carvedilol was more effective at reducing the HVPG. However, the evidence was mostly of low or very low quality, and hence, the findings were uncertain. Later, a retrospective study by Jachs et al. comparing carvedilol and propranolol for secondary prophylaxis of EVH [20] observed lower rates of recurrent EVH, (, (*P* = 0.027), and development/worsening of ascites (*P* = 0.012) in the carvedilol group. This was attributed to a more marked reduction in HVPG (median relative decrease for carvedilol, -20% vs -11% for propranolol; *P* = 0.027), and a more chronic HVPG response in the carvedilol group (53.3% vs 28.6%; *P* = 0.034). In a similar line, a randomized clinical trial assessed the impact of switching to carvedilol 12.5 mg versus remaining on propranolol in patients with cirrhosis and a history of EVH and grade II/III nonrefractory ascites [32]. Ninety-six patients were included, and 64 were switched to carvedilol while 32 were maintained on propranolol. The trial revealed significant improvement in renal function (improvement of glomerular filtration rate: 87.3 ± 2.7 vs. 78.7 ± 2.3 mL/min; *P* = 0.03) at 12-month follow-up, a lower 2-year further decompensation rate (10.5% vs. 35.9%; *P* = 0.003), and a higher 2-year survival rate (86% vs. 64.1%; *P* = 0.01) in the carvedilol group [22]. Importantly, a retrospective multicenter study involving 284 patients with decompensated cirrhosis over a median follow-up of 36.3 months showed a significant reduction in the combined endpoint of further decompensation/death, almost half the risk, in the carvedilol group compared to the cNSBBs group (sub-hazard ratio 0.57; 95% CI 0.42–0.77; *P* < 0.0001) [21]. Our findings build on this evidence, demonstrating not only a lower rate of recurrent EVH with carvedilol use, but also lower rates of other critical outcomes such as ascites, SBP, HRS, HCC, and overall mortality, suggesting that this NSBB helps prevent bleeding and non-bleeding decompensation in patients with cirrhosis and history of EVH. In contrast, a long-term follow-up post hoc analysis of a randomized controlled trial that compared carvedilol and propranolol for reducing HVPG following acute variceal bleeding showed comparable long-term clinical, survival, and safety outcomes over 6 years (except ascites) for these two drugs in patients with decompensated disease [33]. However, the authors note that their small sample size of only 48 select patients for whom the hepatic portal vein gradient response had been titrated may have been a confounding factor, among others.

It is noteworthy to mention that, in addition to being more effective than propranolol, it appears that carvedilol has a better tolerance than therapeutic doses of propranolol titrated to heart rate [14, 32]. This is likely because, at low doses, carvedilol is unlikely to cause hypotension and that it decreases HVPG significantly more than propranolol, and only causes a moderate decrease in cardiac output, heart rate, and blood pressure [14]. This was further showcased in our study by highlighting that carvedilol use was associated with a significantly lower risk of HRS compared to propranolol (RR: 0.677, *P* < 0.001) reflecting its favorable hemodynamic effects. Our findings collectively with those of Kalambokis [32] and Fortea [21] challenge previous safety concerns related to the use of carvedilol in patients with cirrhosis [23, 24]. These findings also agree with the findings by Sinha et al. [34] who found that chronic use of low-dose of carvedilol in patients with mild ascites was associated with improved mortality, compared to patients not on carvedilol, while it did not increase mortality in those with moderate-severe ascites.

Relative to previous studies, our study evaluated a large propensity-matched patient population and is therefore among the most comprehensive analyses on this subject to date. Our study’s extended follow-up period of up to 5 years added valuable longitudinal insights. Furthermore, while most previous studies have often focused on specific outcomes such as EVH, our analysis encompassed a broader range of clinical outcomes. Another strength of this study resides in its use of global, real-world big data from the TriNetX network, which provides a rich dataset encompassing a wide range of demographic and clinical variables, which enhances the generalizability and applicability of the findings. Moreover, the well-defined cohort and robust PSM minimize the impact of confounding variables, enhancing validity.

However, our study has some limitations. First of all, conclusions for guiding clinical practice should not be drawn from observational studies, but rather from randomized controlled trials. Second, although robust PSM was applied to balance the baseline characteristics of the two study groups, some residual confounding factors, such as comorbidities and medications not included in the PSM, may still have been present. Also, the study lacks clinical data that grade disease severity and rather relies on ICD-10 codes diagnoses and matching patients based on baseline comorbidities and MELD laboratory results, which may not fully correspond to clinical conditions. Third, while our data further supports the mounting evidence advocating for carvedilol as the NSBB of choice in the treatment of portal hypertension, our study does not address this question in patients with circulatory and renal dysfunction or recurrent/refractory ascites. Fourth, while the primary objective was to evaluate the risk of outcomes associated with carvedilol use compared to propranolol use, the claims data in this database do not allow for reliable confirmation of medication doses, time interval between EVH diagnosis and medication initiation, medication adherence or therapy-related adverse events. Finally, while HE shares major pathophysiologic mechanisms with ascites and EVH, our analysis did not show additional benefits of carvedilol in reducing HE compared to propranolol; the reason behind which remains unclear to us. In addition to that, HE diagnosis can often be subjective, further adding to this uncertainty.

Future prospective controlled studies that consider disease severity and other clinical features, like etiology of cirrhosis and the presence of other comorbid conditions affecting outcomes, are needed to clarify the impact of specific NSBB on preventing serious outcomes and mortality.

## Conclusion

We showed that carvedilol may have significant benefits over propranolol for secondary prophylaxis of EVH, as well as for mitigating the risk of ascites, SBP, HRS, HCC and overall mortality in patients with cirrhosis and a history of EVH. Furthermore, this study highlights the favorable safety and tolerance profile of carvedilol as reflected by the lower risk of HRS in the carvedilol group which likely reflects more favorable hemodynamic effects. These findings strongly support the selection of carvedilol as the preferred NSBB in this patient population.

## Supplementary Information

Below is the link to the electronic supplementary material.

Supplementary file1 (DOCX 17 KB)

## Data Availability

No datasets were generated or analyzed during the current study.

## Abbreviations

AASLD: American Association for the Study of Liver Diseases
ALD: Alcohol-related Liver Disease
BMI: Body Mass Index
CO: Cardiac Output
CI: Confidence Interval
cNSBBs: Classic non-Selective Beta-Blocker
EASL: European Association for the Study of the Liver
EVL: Esophageal Variceal Ligation
EVH: Esophageal Variceal Hemorrhage
GI: Gastrointestinal
HE: Hepatic Encephalopathy
HCC: Hepatocellular Carcinoma
HRS: Hepatorenal Syndrome
HVPG: Hepatic Venous Pressure Gradient
ICD-10-CM: International Classification of Disease, Tenth Revision, and Clinical Modification
INR: International Normalized Ratio
MAP: Mean Arterial Pressure
MASLD: Metabolic dysfunction-associated Steatotic Liver Disease
MELD: Model for End-Stage Liver Disease
NSBB: Non-Selective Beta-Blocker
PSM: Propensity Score Matching
RR: Relative Risk
SBP: Spontaneous Bacterial Peritonitis

## References

[1] Ginès P, Krag A, Abraldes JG, Solà E, Fabrellas N, Kamath PS. Liver cirrhosis. *The Lancet.* 2021;398:1359–1376. 10.1016/S0140-6736(21)01374-X. 10.1016/S0140-6736(21)01374-X 34543610

[2] Kumar R, Kumar S, Prakash SS. Compensated liver cirrhosis: Natural course and disease-modifying strategies. *World J Methodol.* 2023;13:179–193. 10.5662/wjm.v13.i4.179. 37771878 10.5662/wjm.v13.i4.179 PMC10523240

[3] Mansour D, McPherson S. Management of decompensated cirrhosis. *Clinical Medicine.* 2018;18:s60–s65. 10.7861/clinmedicine.18-2-s60. 29700095 10.7861/clinmedicine.18-2s-s60 PMC6334027

[4] Schwarz M, Schwarz C, Burghart L et al. Late-stage presentation with decompensated cirrhosis is alarmingly common but successful etiologic therapy allows for favorable clinical outcomes. *PLoS ONE.* 2023;18:e0290352. 10.1371/journal.pone.0290352. 37616205 10.1371/journal.pone.0290352 PMC10449133

[5] Sharma M, Rameshbabu CS. Collateral Pathways in Portal Hypertension. *J Clin Exp Hepatol.* 2012;2:338–352. 10.1016/j.jceh.2012.08.001. 25755456 10.1016/j.jceh.2012.08.001 PMC3940321

[6] Iwakiri Y. Pathophysiology of Portal Hypertension. *Clinics in Liver Disease.* 2014;18:281–291. 10.1016/j.cld.2013.12.001. 24679494 10.1016/j.cld.2013.12.001 PMC3971388

[7] Lebrec D, Corbic M, Nouel O, Benhamou JP. PROPRANOLOL—A MEDICAL TREATMENT FOR PORTAL HYPERTENSION? *The Lancet.* 1980;316:180–182. 10.1016/S0140-6736(80)90063-X. 10.1016/s0140-6736(80)90063-x 6105342

[8] Ge PS, Runyon BA. Treatment of Patients with Cirrhosis. Campion EW, ed. *N Engl J Med.* 2016;375:767–777. 10.1056/NEJMra1504367. 27557303 10.1056/NEJMra1504367

[9] Villanueva C, Albillos A, Genescà J. β blockers to prevent decompensation of cirrhosis in patients with clinically significant portal hypertension (PREDESCI): a randomised, double-blind, placebo-controlled, multicentre trial. *Lancet Lond Engl.* 2019;393:1597–1608. 10.1016/S0140-6736(18)31875-0. 10.1016/S0140-6736(18)31875-0 30910320

[10] Villanueva C, Torres F, Sarin SK et al. Carvedilol reduces the risk of decompensation and mortality in patients with compensated cirrhosis in a competing-risk meta-analysis. *J Hepatol.* 2022;77:1014–1025. 10.1016/j.jhep.2022.05.021. 35661713 10.1016/j.jhep.2022.05.021

[11] Villanueva C, Sapena V, Lo GH et al. Improving primary prophylaxis of variceal bleeding by adapting therapy to the clinical stage of cirrhosis. A competing-risk meta-analysis of individual participant data. *Aliment Pharmacol Ther.* 2024;59:306–321. 10.1111/apt.17824. 38108646 10.1111/apt.17824

[12] Sharma M, Singh S, Desai V et al. Comparison of Therapies for Primary Prevention of Esophageal Variceal Bleeding: A Systematic Review and Network Meta-analysis. *Hepatology.* 2019;69:1657–1675. 10.1002/hep.30220. 30125369 10.1002/hep.30220

[13] Rodrigues SG, Mendoza YP, Bosch J. Beta-blockers in cirrhosis: Evidence-based indications and limitations. *JHEP Reports.* 2020;2:100063. 10.1016/j.jhepr.2019.12.001. 32039404 10.1016/j.jhepr.2019.12.001 PMC7005550

[14] Kaplan DE, Ripoll C, Thiele M. AASLD Practice Guidance on risk stratification and management of portal hypertension and varices in cirrhosis. *Hepatol Baltim Md.* 2024;79:1180–1211. 10.1097/HEP.0000000000000647. 10.1097/HEP.0000000000000647 37870298

[15] De Franchis R, Bosch J, Garcia-Tsao G, Reiberger T, Ripoll C, Abraldes JG, Albillos A, Baiges A, Bajaj J, Bañares R, Barrufet M. Baveno VII - Renewing consensus in portal hypertension. *J Hepatol.* 2022;76:959–974. 10.1016/j.jhep.2021.12.022. 35120736 10.1016/j.jhep.2021.12.022 PMC11090185

[16] Bañares R, Moitinho E, Piqueras B et al. Carvedilol, a new nonselective beta-blocker with intrinsic anti-alpha1-adrenergic activity, has a greater portal hypotensive effect than propranolol in patients with cirrhosis. *Hepatology.* 1999;30:79–83. 10.1002/hep.510300124. 10385642 10.1002/hep.510300124

[17] Sinagra E, Perricone G, D’Amico M, Tinè F, D’Amico G. Systematic review with meta-analysis: the haemodynamic effects of carvedilol compared with propranolol for portal hypertension in cirrhosis. *Aliment Pharmacol Ther.* 2014;39:557–568. 10.1111/apt.12634. 24461301 10.1111/apt.12634

[18] Hanif Muhammad Farooq, Fiaz Raja Omer, Iqbal Muhammad Adnan et al. Comparison of carvedilol and propranolol for primary prophylaxis of esophageal variceal bleed in cirrhotic patients. *Pak J Pharm Sci.* 2023;36:857–862. 37580935

[19] Zacharias AP, Jeyaraj R, Hobolth L, Bendtsen F, Gluud LL, Morgan MY. Carvedilol versus traditional, non-selective beta-blockers for adults with cirrhosis and gastroesophageal varices. Cochrane Hepato-Biliary Group, ed. *Cochrane Database of Systematic Reviews*. 2018;2018(10). 10.1002/14651858.CD011510.pub2 10.1002/14651858.CD011510.pub2 PMC6517039 30372514

[20] Jachs M, Hartl L, Simbrunner B. Carvedilol Achieves Higher Hemodynamic Response and Lower Rebleeding Rates Than Propranolol in Secondary Prophylaxis. *Clin Gastroenterol Hepatol Off Clin Pract J Am Gastroenterol Assoc.* 2023;21:2318–2326. 10.1016/j.cgh.2022.06.007. 10.1016/j.cgh.2022.06.007 35842118

[21] Fortea JI, Alvarado-Tapias E, Simbrunner B. Carvedilol vs. propranolol for the prevention of decompensation and mortality in patients with compensated and decompensated cirrhosis. *J Hepatol Published online December*. 17, 2024:S0168–8278(24)02772–7. 10.1016/j.jhep.2024.12.017 10.1016/j.jhep.2024.12.017 39701300

[22] Cheung KS, Mok CH, Lam LK et al. Carvedilol Versus Other Nonselective Beta Blockers for Variceal Bleeding Prophylaxis and Death: A Network Meta-analysis. *J Clin Transl Hepatol.* 2023;11:1143–1149. 10.14218/JCTH.2022.00130S. 37577228 10.14218/JCTH.2022.00130S PMC10412710

[23] Moctezuma-Velazquez C, Kalainy S, Abraldes JG. Beta-blockers in patients with advanced liver disease: Has the dust settled? *Liver Transpl.* 2017;23:1058–1069. 10.1002/lt.24794. 28590564 10.1002/lt.24794

[24] Schwarzer R, Kivaranovic D, Paternostro R et al. Carvedilol for reducing portal pressure in primary prophylaxis of variceal bleeding: a dose-response study. *Alimentary Pharmacology & Therapeutics.* 2018;47:1162–1169. 10.1111/apt.14576. 29492989 10.1111/apt.14576

[25] Reverter E, Tandon P, Augustin S et al. A MELD-based model to determine risk of mortality among patients with acute variceal bleeding. *Gastroenterology.* 2014;146:412-419.e3. 10.1053/j.gastro.2013.10.018. 24148622 10.1053/j.gastro.2013.10.018

[26] Sersté T, Melot C, Francoz C. Deleterious effects of beta-blockers on survival in patients with cirrhosis and refractory ascites. *Hepatol Baltim Md.* 2010;52:1017–1022. 10.1002/hep.23775. 10.1002/hep.23775 20583214

[27] Sersté T, Francoz C, Durand F. Beta-blockers cause paracentesis-induced circulatory dysfunction in patients with cirrhosis and refractory ascites: a cross-over study. *J Hepatol.* 2011;55:794–799. 10.1016/j.jhep.2011.01.034. 21354230 10.1016/j.jhep.2011.01.034

[28] EASL Clinical Practice Guidelines for the management of patients with decompensated cirrhosis. *J Hepatol*. 2018;69(2):406–460. 10.1016/j.jhep.2018.03.024 10.1016/j.jhep.2018.03.024 29653741

[29] Turco L, Villanueva C, La Mura V et al. Lowering Portal Pressure Improves Outcomes of Patients With Cirrhosis, With or Without Ascites: A Meta-Analysis. *Clin Gastroenterol Hepatol.* 2020;18:313-327.e6. 10.1016/j.cgh.2019.05.050. 31176013 10.1016/j.cgh.2019.05.050

[30] Alvarado-Tapias E, Ardevol A, Garcia-Guix M et al. Short-term hemodynamic effects of β-blockers influence survival of patients with decompensated cirrhosis. *J Hepatol.* 2020;73:829–841. 10.1016/j.jhep.2020.03.048. 32298768 10.1016/j.jhep.2020.03.048

[31] Malandris K, Paschos P, Katsoula A et al. Carvedilol for prevention of variceal bleeding: a systematic review and meta-analysis. *Ann Gastroenterol.* 2019;32:287–297. 10.20524/aog.2019.0368. 31040627 10.20524/aog.2019.0368 PMC6479656

[32] Kalambokis GN, Christaki M, Tsiakas I. Conversion of Propranolol to Carvedilol Improves Renal Perfusion and Outcome in Patients With Cirrhosis and Ascites. *J Clin Gastroenterol.* 2021;55:721–729. 10.1097/MCG.0000000000001431. 32991355 10.1097/MCG.0000000000001431

[33] Sharma S, Agarwal S, Gunjan D. Long-term Outcomes with Carvedilol versus Propranolol in Patients with Index Variceal Bleed: 6-year Follow-up Study. *J Clin Exp Hepatol.* 2021;11:343–353. 10.1016/j.jceh.2020.08.009. 33994717 10.1016/j.jceh.2020.08.009 PMC8103346

[34] Sinha R, Lockman KA, Mallawaarachchi N, Robertson M, Plevris JN, Hayes PC. Carvedilol use is associated with improved survival in patients with liver cirrhosis and ascites. *J Hepatol.* 2017;67:40–46. 10.1016/j.jhep.2017.02.005. 28213164 10.1016/j.jhep.2017.02.005

