# Supplementary material for: Comparative Risk of Recurrent Esophageal Variceal Hemorrhage and Other Decompensation Events with Carvedilol Versus Propranolol in Patients with Cirrhosis: A Retrospective Study
Source: Dig Dis Sci. 2025 Oct 18;71(4):1556–68. doi: 10.1007/s10620-025-09453-6 (PMC13144202; doi:10.1007/s10620-025-09453-6)
Supplement: Supplementary file 1 — Supplementary file1 (DOCX 17 KB) [file 10620_2025_9453_MOESM1_ESM.docx]

**Supplemental Table 1**: ICD-10-CM, RxNorm, & CPT Codes used in the analysis

| **Diagnosis/Medications/Procedure** | **ICD-10-CM, RxNorm, & CPT Codes** |
| --- | --- |
| Cirrhosis | K76.4 |
|  | K70.3 |
| Esophageal Variceal Hemorrhage | I85.01 |
|  | I85.11 |
| Hepatic Encephalopathy | K76.82 |
| Ascites | R18 |
| Spontaneous Bacterial Peritonitis | K65.2 |
| Hepatorenal Syndrome | K76.7 |
| Liver Transplant status | Z94.4 |
| Hepatocellular Carcinoma | C22.0 |
| Acute Kidney Failure and Chronic Kidney Disease | N17, N18, N19 |
| Alcohol-related disorders | F10 |
| EGD with EBL | 43244 |
| Carvedilol | 20352 |
| Propranolol | 8787 |
| Ciprofloxacin | 2551 |
| Norfloxacin | 7517 |
| Moxifloxacin | 139462 |
| Ceftriaxone | 2193 |
| Piperacillin/tazobactam | 8339 |
| Trimethoprim/sulfamethoxazole | 10829 |

Abbreviations: EGD, Esophagogastroduodenoscopy; EBL, Esophageal Band Ligation
